# Mammalian PI-Phospholipase C Isozymes: Structural and Functional Insights and Roles in Health and Disease

**DOI:** 10.3390/medicina61061054

**Published:** 2025-06-07

**Authors:** May Hamdi, Mohammed Al-Matwi, Nour Elghoul, Hissa Al-Kuwari, Tahseen S. Sayed, Emna Riguene, Michail Nomikos

**Affiliations:** College of Medicine, QU Health, Qatar University, Doha P.O. Box 2713, Qatar; mh2106279@qu.edu.qa (M.H.); ma2104272@qu.edu.qa (M.A.-M.); ne2106465@qu.edu.qa (N.E.); ha2104524@qu.edu.qa (H.A.-K.); tahseen.s@qu.edu.qa (T.S.S.); emna.riguene@qu.edu.qa (E.R.)

**Keywords:** Phosphoinositide Specific-Phospholipase C Isoforms, catalytic activity, phosphoinositide metabolism, Calcium Signaling, cancer, infertility, therapeutic targets

## Abstract

The Phosphoinositide Specific-Phospholipase C (PI-PLC) family of enzymes plays a crucial role in various cellular processes by catalyzing the hydrolysis of phosphatidylinositol 4,5-bisphosphate [PI(4,5)P_2_] into inositol 1,4,5-trisphosphate (IP_3_) and diacylglycerol (DAG), which are essential messengers mediating critical intracellular signaling pathways. Herein, we carry out a comprehensive analysis of the structure, function, regulation, and implications of the PI-PLC family enzymes in both physiological and pathological contexts. More specifically, we discuss the structural features of PI-PLCs, elucidating their conserved domains and catalytic mechanisms. Furthermore, we explore the multifaceted roles of PI-PLCs in signal transduction, cellular homeostasis, and membrane dynamics, whilst highlighting the intricate regulatory mechanisms governing their activity such as protein–protein interactions, post-translational modifications, and lipid modulation. Lastly, we assess the involvement of PI-PLCs in various diseases, such as cancer, neurological disorders, immune dysregulation, and male infertility, emphasizing their potential as therapeutic targets.

## 1. Introduction

Glycerophospholipids are a group of phospholipids comprising a hydrophilic phosphate head and two hydrophobic fatty acid tails attached to a glycerol backbone [1]. Due to their amphipathic nature, glycerophospholipids are integral components of membranes in all living organisms accounting for 50–60% of membrane lipid content [2]. As such, they separate and protect the cells and the subcellular organelles from their external environments, thus enabling compartmentalization and homeostasis. However, cell and subcellular membranes across different cell types are characterized by diversity and lateral asymmetry in glycerophospholipid distribution [3]. This heterogeneity leads to differential physiological functions, from membrane trafficking to cell signaling, that are regulated by lipid-metabolizing enzymes [4].

The hydrolysis of phospholipids by a class of enzymes called phospholipases is fundamental to initiating a cascade of signaling events that govern cell membrane dynamics and crucial cellular processes [5]. Based on the specific bond they hydrolyze within the phospholipid molecule, phospholipases are categorized into Phospholipase A (PLA), Phospholipase B (PLB), Phospholipase C (PLC), and Phospholipase D (PLD). Even though PLA, PLB, and PLD are particularly important for lipid metabolism and structural remodeling of membranes, PLC is indispensable due to its role in signal transduction pathways that are critical for cellular processes such as cell differentiation, immune responses, and cancer regulation [6,7,8,9,10].

PLC is a family of enzymes with a phosphodiesterase function catalyzing the hydrolysis of glycerophospholipids. Mammalian PLCs can be classified based on their preferred glycerophospholipid substrate into three subtypes: PI-PLC, phosphatidylcholine-specific PLC (PC-PLC) and phosphatidylethylamine-specific PLC (PE-PLC). Even though the activities of mammalian PC-PLC and PE-PLC have been perennially reported, the proteins themselves and their corresponding genes remain unidentified [11]. On the contrary, PI-PLC has been extensively studied and has been shown to hydrolyze membrane-bound phosphoinositides. There are seven biologically distinct phosphoinositide species—PI(3)P, PI(4)P, PI(5)P, PI(3,4)P_2_, PI(4,5)P_2_, PI(3,5)P_2_, and PI(3,4,5)P_3_—generated by phosphorylation of the inositol ring of phosphatidylinositol.

While PI-PLCs are classified as phosphoinositide-specific, they predominantly hydrolyze phosphatidylinositol 4,5-bisphosphate [PI(4,5)P_2_] into two second messengers, diacylglycerol (DAG) and inositol 1,4,5-trisphosphate (IP_3_), which then activate protein kinase C (PKC) and the IP_3_ receptor involved in the release of Ca^2+^ from the endoplasmic reticulum into the cytoplasm. This dual messenger system integrates extracellular signals into intracellular responses activating cell signaling pathways, thus serving several cellular functions [12].

It is hypothesized that all PI-PLCs originated from a single archetype and later diversified as animal species evolved [13]. Mammalian PI-PLC traditionally has six main subfamilies—β, γ, δ, ε, η, and ζ—classified based on commonalities in their activation mechanism, size, amino acid sequence, and structure [14]. There is no PI-PLCα because the protein originally identified as the α isoform was later found to be a disulfide isomerase that lacks phospholipase activity [15]. Each of the aforementioned subfamilies contains one or more members, leading to 13 well-characterized isozymes. However, recent studies have identified three additional atypical isozymes (PLC-XDI, II, III), containing a single X domain, bringing the total number of PI-PLC isozymes to sixteen, although these new isozymes are not yet fully characterized or universally accepted [16].

Additionally, alternative splicing of the genes that encode for the different PI-PLC isoforms gives rise to several splice variants, allowing for more than 20 distinct PI-PLC polypeptide sequences to exist. Alternative splicing enhances the already profound diversity of the members of the PI-PLC family, since splice variants can differ in their structural components, regulation, cellular functions, and localization within tissues and cells [17].

The PI-PLC family’s diversity—spanning subfamilies, isoforms, and splice variants—positions it among the most versatile enzyme families, and the distribution of PI-PLC isoforms across tissues and organelles spatiotemporally regulates Ca^2+^ signaling and gene expression.

## 2. Structural Motifs of PI-PLCs

The majority of PI-PLC isozymes share the same ordered arrangement of certain protein domains. Protein domains are independently folding and often functionally distinct structural units within a protein. Typically, a PI-PLC isoform consists of an N-terminal Pleckstrin homology (PH) domain followed by an EF-hand domain, an X and Y catalytic domain split by an X-Y X-Y linker region and finally a C2 domain at the C-terminus.

**Figure 1:**
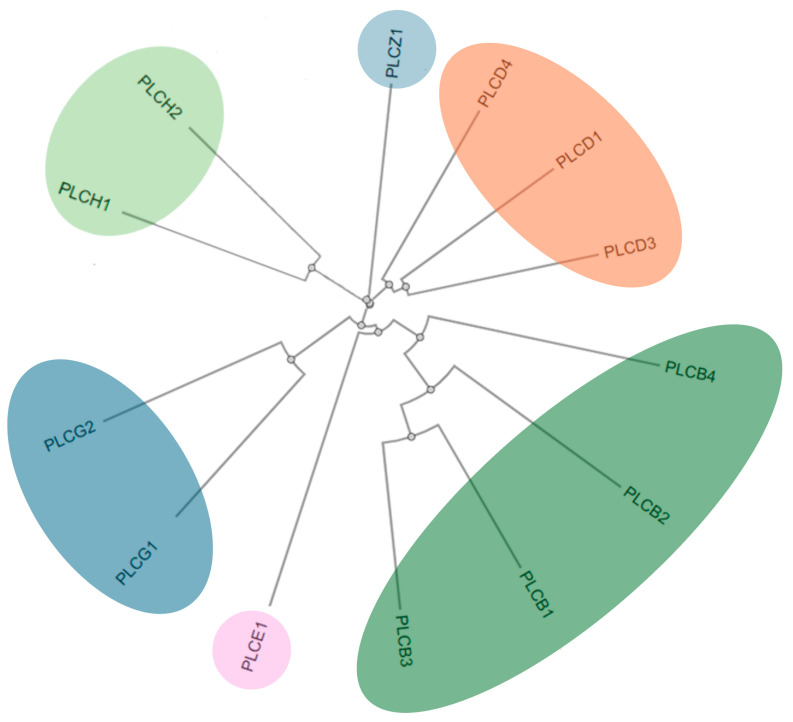
Phylogenetic tree of PI-PLC isozymes generated with Clustal-W. PI-PLCβ subfamily is represented in dark green, PI-PLCγ subfamily is represented in dark blue, PI-PLCδ subfamily is represented in orange, PI-PLCε subfamily is represented in pink, PI-PLCζ subfamily is represented in light blue and PI-PLCη subfamily is represented in light green.

Even though they share the same domain organization, the sequence similarity for specific domains is highly variable among different isozymes. The residues in the PH domains that mediate ligand binding are poorly conserved among different PI-PLC isozymes, leading to functional diversity between the PH domains of different PI-PLCs. The catalytic XY domain is the only notable exception to this heterogeneity, since the sequence similarity in the X and Y domains is ~60% among all 13 mammalian isozymes, and it is substantially greater among members of the same PI-PLC subfamily. These patterns underlies both the common catalytic function and the regulatory diversity observed among PLC isozymes (Figure 1) [18].

**Figure 2:**
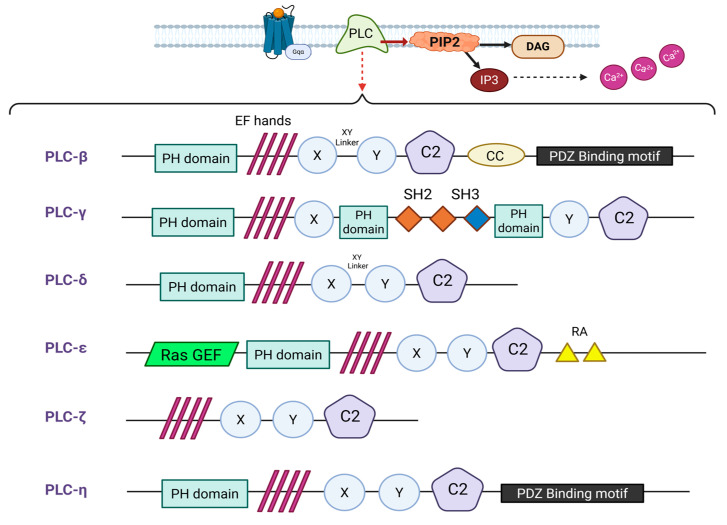
Domain organization shared across the mammalian PI-PLC isoforms. Created in BioRender. Nomikos, M. (2025). https://BioRender.com/a63ns1m (accessed on 26 May 2025).

There are also some deviations from the domain organization pattern with the absence of a PH domain from PI-PLCζ amino acid sequence to be the most distinctive one. Other deviations are the presence of a PDZ domain at the C-terminus of PI-PLCβ and PI-PLCη isozymes, another split pleckstrin homology (PH) domain interrupted by Src homology 2 (SH2) and Src homology 3 (SH3) domains in PI-PLCγ isozymes and an aminoterminal CDC25 homology domain plus two carboxyterminal RA domains in PI-PLC isozymes (Figure 2) [19].

### 2.1. PH Domain

Pleckstrin homology (PH) domains of PI-PLCs are lipid-binding structural motifs facilitating interactions with phosphoinositides in biological membranes. They share very low sequence identity (<20%) but high structural similarity [20]. It is located at the N-terminus of most PI-PLCs and consists of seven β-strands (β1-β7) that form two antiparallel sheets adopting a β-sandwich fold and a single C-terminal *α*-helix [21]. The PH domain of PI-PLCs possesses strong electrostatic polarization, which facilitates interactions with negatively charged phospholipids [22].

The PH domain of PI-PLCβ exhibits weak, nonspecific lipid binding and instead stabilizes intraprotein contacts with EF hands and the catalytic XY domain [23]. The β1-β2 and β3-β4 loops of the PI-PLCγ PH domain in PI-PLCγ features a split architecture interrupted by SH2/SH3 domains, enabling dual regulation by phosphatidylinositol 3,4,5-trisphosphate (PIP_3_) and tyrosine phosphorylation, which is critical for growth factor signaling and forming a binding pocket for phosphoinositides [24]. The PH domain in several PI-PLC isoforms interact with PI(4,5)P_2_ through conserved basic residues, supporting membrane localization necessary for substrate accessibility [25].

The only PI-PLC isozyme that lacks a PH domain from its sequence is PI-PLCζ. The role of the PH domain in this protein seems to be compensated by the positively charged N-terminal lobe EF-hand domain along with the basic amino acids at the C-terminal end of its X-Y linker region, which has been shown to play an important role in PI-PLCζ interactions with PI(4,5)P_2_ [26].

### 2.2. EF-Hand Domain

The EF-hand domain is a structurally conserved Ca^2+^ -binding module critical for enzymatic stability, membrane interactions, and catalytic regulation. It comprises four helix–loop–helix motifs arranged in two lobes (EF1/EF2 and EF3/EF4). These two lobes are not structurally or functionally equivalent. The first lobe (EF1/EF2) supports enzymatic activity, while the second lobe (EF3/EF4) is essential for Ca^2+^ sensitivity and structural stabilization of the catalytic core. Truncation of EF1/EF2 in PI-PLCζ reduces PI(4,5)P_2_ hydrolysis and impairs Ca^2+^-oscillation induction in eggs, while deletion of the second lobe (EF3/EF4) in PI-PLCδ1 inactivates the enzyme by compromising enzyme’s structural integrity [27].

In contrast to other EF-hand-bearing proteins, EF-hand domains in PI-PLCs lack conserved Ca^2+^-binding residues in their loops, particularly in EF3 and EF4. PI-PLCδ1 does not bind Ca^2+^ at its EF hands, as confirmed by crystallographic and solution studies, with Ca^2+^ binding instead localized to the catalytic domain and C2 domain [28]. The EF-hand domain also facilitates membrane targeting, since cationic residues in the EF hands of PI-PLCζ interact with anionic phospholipids, compensating for the absence of a PH domain [26]. The unique adaptation of EF-hand domains in PI-PLCs shows that their roles have evolved beyond classical Ca^2+^ binding to include enzyme activity regulation and substrate interaction.

### 2.3. XY Catalytic Domain

The XY domain is structurally conserved across PI-PLCs, reflecting its indispensable role in signal transduction. The XY catalytic domain constitutes the enzymatic core of PI-PLCs and adopts a distorted triose phosphate isomerase (TIM) barrel fold. The X domain houses the active site residues critical for catalysis and the Y domain contributing to substrate binding and structural stabilization [29]. Together, they form a deep catalytic cleft that accommodates PI(4,5)P_2_, enabling precise cleavage of its phosphodiester bond and allowing integration of diverse regulatory inputs across isoforms [30].

The X domain contains conserved histidine and glutamate residues, which coordinate Ca^2+^ ions and facilitate nucleophilic attack on the phosphodiester bond of the substrate. The Y domain stabilizes interactions with the inositol headgroup of PI(4,5)P_2_ through electrostatic contacts, ensuring substrate specificity over other structurally related lipids like phosphatidylinositol (PI) or phosphatidylinositol phosphate (PIP) [30].

A flexible, charged linker between the X and Y domains, termed the X-Y linker, serves as a regulatory switch, obstructing substrate access in the inactive state. Activation mechanisms—such as Gαq binding to PI-PLCβ or tyrosine phosphorylation of PI-PLCγ—displace the X-Y linker, relieving autoinhibition and permitting catalytic activity. On the contrary, unlike somatic PLCs, the absence of the PLCζ X-Y linker significantly diminishes both in vitro PI(4,5)P_2_ hydrolysis and in vivo Ca^2+^-oscillation-inducing activity, revealing evidence for a novel PLCζ enzymatic mechanism [31,32].

Distinct regulatory domains appended to the XY domain enable PI-PLC isoforms to respond to diverse signaling cues. PI-PLCβ isoforms are activated by direct binding of Gαq subunits to their C-terminal tail, positioning the catalytic core near membrane-bound PI(4,5)P_2_. PI-PLCγ isoforms rely on SH2 domains to translocate to tyrosine-phosphorylated receptors, where their X-Y domains gain access to the substrate. PI-PLC-ε integrates inputs from small GTPases like Ras, which modulate catalytic efficiency through allosteric interactions [12].

### 2.4. C2 Domain

The C2 domain is a structurally conserved module, primarily mediating membrane interactions in a Ca^2+^-dependent manner. Located at the C-terminus of all PI-PLCs, this domain adopts an eight-stranded antiparallel β-sandwich fold with three Ca^2+^-binding loops (CBR1–CBR3) at one end, critical for membrane association and regulatory crosstalk [33].

Ca^2+^ binding induces conformational changes that expose hydrophobic residues, facilitating transient interactions with anionic phospholipids. While the PI-PLCδ1 C2 domain binds Ca^2+^, its membrane affinity is weaker than that of other C2-containing proteins. On the contrary, the PI-PLCδ4-C2 domain has a stronger Ca^2+^-independent membrane affinity, arising from the presence of positively charged residues (e.g., lysine, arginine) in their Ca^2+^-binding loops. These residues enable electrostatic interactions with anionic phospholipids (e.g., phosphatidylserine, phosphoinositides) even in the absence of Ca^2+^. PI-PLCδ4 can pre-bind diverse membranes before Ca^2+^ influx [34].

In PI-PLCδ1, the C2 domain stabilizes the catalytic core by forming a rigid interface with the EF-hand domain, whereas in PI-PLCβ and γ, the C2 domain stabilizes the catalytic core indirectly via protein–protein interactions (Gαq or cSH2). Loss of the C2 domain disrupts enzymatic activity across isoforms, highlighting its conserved role in maintaining catalytic competence [35].

The conserved domain organization of PI-PLC isozymes demonstrates their dynamic involvement in regulating enzymatic activity and precise response to cellular signals. While the PH domain binds to PI(4,5)P_2_ and enables membrane localization by positioning the enzyme near the lipid substrate, the EF-hand domain confers structural stability and contributes towards enzymatic activation due to its role in modulating Ca^2+^ sensitivity. The XY catalytic domain is responsible for generating IP_3_ and DAG. The C2 domain along with the maintenance of structural integrity also supports membrane interactions. Lastly, the X-Y linker acts in most cases as an autoinhibitory region. This intricate framework ensures that the enzymatic activity of PI-PLCs is initiated under appropriate conditions.

## 3. Structure, Localization, and Tissue Distribution of PI-PLC Isozymes

Despite their common core domain organization and widespread cellular coexpression, PI-PLC isoforms exhibit distinct structures, tissue expression patterns, and subcellular localizations, which critically define their signaling roles [12].

### 3.1. PI-PLCβ

The mammalian PI-PLCβ subfamily includes four isozymes—PI-PLCβ1, PI-PLCβ2, PI-PLCβ3, and PI-PLCβ4—that are activated by heterotrimeric G proteins associated with G protein-coupled receptors (GPCRs). All four PI-PLCβ isozymes are directly activated by the Gαq subunit following GPCR stimulation. Additionally, PI-PLCβ2 and PI-PLCβ3 are strongly activated by Gβγ dimers released from G proteins, while PI-PLCβ1 and PI-PLCβ4 can also weakly respond to Gβγ [36].

Each of the PI-PLCβ1, PI-PLCβ2, and PI-PLCβ4 isozymes has splice variants. More specifically, the human PI-PLCβ1 gene has two alternative splice variants, PI-PLCβ1a and PI-PLCβ1b, differing at the extreme C terminus, where the 75 residues of PI-PLCβ1a carboxyterminal sequence are replaced by a shorter sequence of 32 residues in the PI-PLCβ1b splice variant [37,38]. The more prevalent PI-PLCβ1a contains a PDZ-interacting sequence at the C-terminus, whereas the carboxyterminal sequence of PI-PLCβ1b exhibits proline-rich domains that are responsible for targeting the membrane by association with the scaffolding protein Shank3 [39]. Similarly, human PI-PLCβ2 also has two splice variants, PI-PLCβ2a and PI-PLCβ2b, differing at the extreme C terminus, where the PI-PLCβ2b splice variant is 15 residues shorter at the C-terminus than the PI-PLCβ2a splice variant [40]. Furthermore, the PI-PLCβ4 gene has three splice variants. Variant 1 lacks an internal segment and possesses a longer, distinct C-terminus. The other variants differ by the inclusion or exclusion of specific exons, with at least one representing the longest transcript [41].

PI-PLCβ isozymes share a conserved catalytic core comprising several domains. The PH domain exhibits weak lipid binding and stabilizes intraprotein interactions with EF hands and the XY catalytic domain. The EF-hand domain supports Gαq binding and stabilizing the catalytic core but lacks Ca^2+^-binding residues. The XY catalytic domain houses the active site for PI(4,5)P_2_ hydrolysis, with the X-Y linker playing an autoinhibitory role along with the autoinhibitory helix (Hα’) at the proximal C-terminal domain (CTD). The C2 domain is involved in Ca^2+^-dependent membrane binding and regulatory interactions with the EF hands and distal CTD [20].

The C-terminal extension is a unique structural feature of PI-PLCβ isozymes. It includes a proximal CTD containing the primary Gαq-binding site and the Hα’ that interacts with the catalytic core, preventing enzymes’ overactivity in the absence of activation signals. The distal CTD forms a coiled-coil helical bundle that mediates membrane binding via interactions with anionic lipids, thus facilitating access to the phospholipid substrate [20]. The C-terminal extension provides specialized mechanisms for direct activation by G proteins, strong membrane association, and autoinhibitory regulation. These features allow PI-PLCβ isozymes to serve as critical effectors in GPCR signaling pathways, distinguishing them functionally and structurally from other PI-PLC subfamilies [42].

PI-PLCβ isozymes show varied expression. PI-PLCβ1 and PI-PLCβ3 are ubiquitously expressed, while PI-PLCβ2 is restricted to hematopoietic cells (e.g., immune cells), and PI-PLCβ4 is enriched in neurons and macrophages. PI-PLCβ1 is predominantly membrane-bound due to strong distal CTD–lipid interactions and has splice variants that alter Gαq coupling and subcellular localization. PI-PLCβ2 is restricted to hematopoietic cells (e.g., neutrophils, macrophages) and is preferentially activated by Gβγ subunits and Rac GTPases. PI-PLCβ3 is primarily cytosolic but translocates to membranes upon Gαq binding and is the only isoform with a full-length crystal structure, revealing a curved distal CTD resembling BAR domains. PI-PLCβ4 is enriched in Purkinje cells and the visual cortex, critical for synaptic plasticity (Table 1) [20].

### 3.2. PI-PLCγ

The mammalian PI-PLCγ subfamily includes two isozymes—PI-PLCγ1 and PI-PLCγ2—that share a conserved catalytic core with the other PI-PLC isozymes. The N-terminal PH domain contributes to membrane localization, protein–protein interactions with cytoskeletal elements, and the regulation of PI-PLCγ activity. The catalytic core comprises X and Y domains, forming a distorted TIM barrel responsible for PI(4,5)P_2_ hydrolysis. The EF-hand and C2 domains stabilize the catalytic core and contribute to Ca^2+^-dependent membrane interactions, though the C2 domain of PI-PLCγ lacks functional Ca^2+^-binding sites [20].

Even though the domain organization of PI-PLCγ isozymes is similar to that of the rest of the PI-PLCs, the X-Y linker is uniquely differentiated. It comprises Src homology 2 (SH2) and Src homology 3 (SH3) domains embedded in a split PH domain. The PH domain is divided into two segments (PH-N and PH-C) flanking the SH2/SH3 region, with the PH-C domain binding PIP_3_ to facilitate membrane recruitment in PI3K-activated pathways. The SH2 domain binds to phosphorylated tyrosine residues on receptor tyrosine kinases (RTKs) or adaptor proteins (e.g., LAT), enabling the activation of downstream signaling proteins by tyrosine kinases, while the SH3 domain interacts with proline-rich motifs in cytoskeletal regulators (e.g., dynamin, WASP) to link PI-PLCγ to membrane remodeling. Moreover, the C-terminal SH2 domain within the X-Y linker is the critical determinant for autoinhibition of PI-PLCγ activity [43]. These distinct features of the PI-PLCγ X-Y linker enable direct, high-affinity interactions with activated receptors and adaptors, scaffold signaling complexes, and provide unique regulatory control over enzyme activity—features that are absent in other PI-PLC isoforms [20].

PI-PLCγ1 is ubiquitously expressed and critical for growth factor signaling (e.g., EGF, NGF) and cell proliferation, with nuclear localization enabling DAG generation to regulate transcription factors like NF-κB via PKC. PI-PLCγ2 is enriched in immune cells (B cells, mast cells, neutrophils) and essential for FcεR and BCR signaling, directly binding active Rac GTPase via its split PH domain to enable membrane targeting independent of tyrosine phosphorylation [20].

Tyrosine phosphorylation is a primary activation mechanism: RTKs (e.g., EGFR) phosphorylate PI-PLCγ1 at Y783 and Y1254, relieving autoinhibition and enhancing catalytic activity, while immune receptors (e.g., FcεR) phosphorylate PI-PLCγ2 at Y753 and Y759 to promote membrane recruitment. PIP_3_ binding to the PH-C domain recruits PI-PLCγ to PI3K-rich membranes, such as growth factor-stimulated ruffles, and potentiates PI(4,5)P_2_ hydrolysis through SH2–phosphotyrosine interactions. The SH3 domain binds cytoskeletal effectors like dynamin to regulate endocytosis and podosome formation, while PI-PLCγ2 directly engages Rac-GTP via its PH domain, bypassing tyrosine kinase requirements in macrophages [44].

### 3.3. PI-PLCδ

The PI-PLCδ family comprises three isoforms widely distributed across several tissues. These are δ1, δ3, and δ4. These isoforms share conserved domains and lack distinctive domains, indicating that PI-PLCδ may serve as the foundation for other PI-PLC types [45]. PI-PLCδ isozymes show greater sensitivity to Ca^2+^ [13]. The PI-PLCδ1 gene has two splicing variants, PI-PLCδ1a and PI-PLCδ1b, differing by 274 amino acid residues [46].

PI-PLCδ isoforms share a conserved domain architecture, including a PH domain that binds PI(4,5)P_2_ with nanomolar affinity via basic lysine residues, anchoring the enzyme to the plasma membrane. The EF-hand domain stabilizes the catalytic core but lacks Ca^2+^-binding capacity, distinguishing them from canonical EF-hand proteins. The catalytic TIM barrel, split into X and Y regions, forms the active site for PI(4,5)P_2_ hydrolysis. The C2 domain coordinates Ca^2+^ ions via three loops (CBR1–CBR3) and interacts with anionic lipids, enhancing membrane residence time under elevated Ca^2+^ conditions.

PI-PLCδ1 is ubiquitously expressed and serves as the primary regulator of basal PI(4,5)P_2_ turnover in most tissues, exhibiting high Ca^2+^ sensitivity through its C2 domain, which binds 2–3 Ca^2+^ ions (Kd ~10–100 μM) to facilitate membrane recruitment during Ca^2+^ spikes. PI-PLCδ3 is enriched in the brain and secretory tissues, regulating synaptic vesicle release and exocytosis, with splice variants that modify EF-hand interactions to alter Ca^2+^ dependence. PI-PLCδ4 is testis-specific, critical for spermatogenesis and sperm motility, and includes truncated isoforms lacking functional PH domains that retain catalytic activity [46].

Ca^2+^-dependent activation occurs through Ca^2+^ binding to the C2 domain, which exposes hydrophobic residues to enable transient membrane interactions. PI-PLCδ1’s activity increases 10-fold at cytosolic Ca^2+^ concentrations exceeding 300 nM. Membrane tethering is mediated by the PH domain’s high PI(4,5)P_2_ affinity, ensuring constitutive membrane association for processive PI(4,5)P_2_ hydrolysis. Mutations in the PH domain reduce membrane binding and impair signaling. Autoregulation involves IP_3_ competing with PI(4,5)P_2_ for PH domain binding, creating a feedback loop to limit excessive signaling [46].

### 3.4. PI-PLCε

Two forms of PI-PLCε have been found to arise from alternative splicing at the aminoterminal end with no functional differences. These are designated as PI-PLCε1a and PI-PLCε1b, and they differ in size by 25 kDa [47]. PI-PLCε contains a modular architecture that sets it apart from other PI-PLC families. Apart from a PH domain, four EF-hand motifs, a split catalytic X-Y domain, and a C2 domain, PI-PLCε possesses a unique CDC25 homology domain at the N-terminus that functions as a GEF for Ras and Rap1 GTPases, enabling PI-PLCε to amplify its own activation through feedback loops. There are also two distinct Ras association (RA) domains (RA1 and RA2) at the C-terminus mediating interactions with active Ras and Rap1: RA1 integrates with the catalytic core, while RA2 binds Rap1-GTP to sustain signaling. The X-Y linker contains an amphipathic helix that autoinhibits basal activity. A unique C-terminal region facilitates interactions with RhoA and other regulators [48].

PI-PLCε is activated through multiple pathways. GTPase interactions, including Ras and Rap1 interactions, recruit PI-PLCε to the plasma membrane (via Ras) or perinuclear regions such as the Golgi (via Rap1). RhoA directly binds PI-PLCε’s C-terminal region, enhancing catalytic activity. GPCRs coupled to Gα12/13 or Gαq activate PI-PLCε via RhoA or Ras, respectively. Autoregulation occurs through the X-Y linker’s amphipathic helix, which suppresses basal activity; GTPase binding relieves this inhibition. PI-PLCε exists as a single isoform in mammals but exhibits splice variants influencing localization and activity. The CDC25 domain is essential for sustained Rap1 activation and Golgi retention, while the RA2 domain mediates feedback loops with Rap1-GTP [45,47].

### 3.5. PI-PLCζ

**Figure 3:**
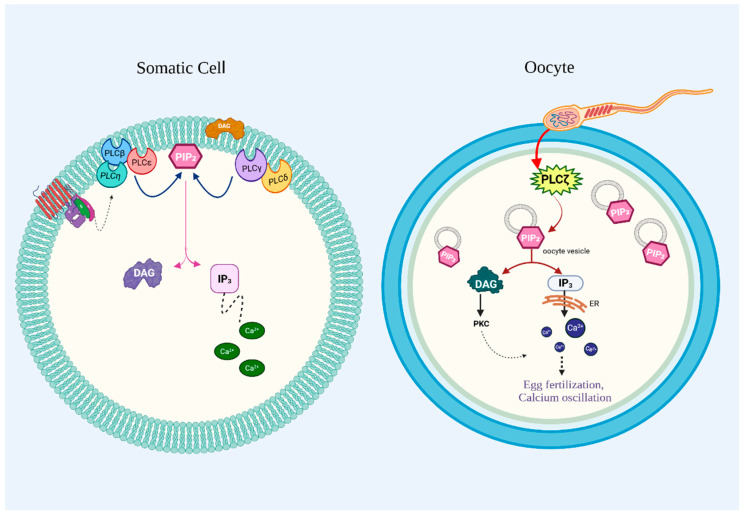
Differential substrate targeting for PI-PLCζ compared to the other PI-PLC isoforms. PI-PLCζ targets intracellular pools of PI(4,5)P_2_, whereas all other PI-PLC isoforms target membrane-bound PI(4,5)P_2_. Created in BioRender. Nomikos, M. (2025). https://BioRender.com/m8w6veh (accessed on 26 May 2025).

PI-PLCζ contains a minimalistic domain architecture compared to the other PI-PLC isozymes since it lacks a PH domain from its sequence. The absence of a PH domain is compromised by the presence of a positively charged X-Y linker that allows PI-PLCζ to bind PI(4,5)P_2_ on intracellular membranes bypassing plasma membrane localization seen in other PI-PLCs (Figure 3).

The EF-hand domain at the N-terminus binds Ca^2+^ and contributes to PI(4,5)P_2_ substrate interaction. The XY catalytic domain forms the active site for PI(4,5)P_2_ hydrolysis. The X-Y linker contains a positively charged segment critical for membrane targeting via electrostatic interactions with anionic PI(4,5)P_2_. The C2 domain, at the C-terminus, stabilizes membrane binding but lacks functional Ca^2+^-binding sites [49].

PI-PLCζ is maintained in an inactive state within sperm, requiring oocyte-specific factors for activation post-fertilization. Proteolytic cleavage of sperm PI-PLCζ generates functional heterodimers with enhanced activity, as observed in mouse and porcine models [50]. Moreover, mouse PI-PLCζ has been shown to contain a functional NLS enabling nuclear translocation [51].

PI-PLCζ is exclusively expressed in the testis. Furthermore, it triggers Ca^2+^ oscillations only in oocytes and not in somatic cells [52]. Clinical studies directly link PI-PLCζ mutations to male infertility, while biochemical studies emphasize its dependence on oocyte factors for activation [53].

### 3.6. PI-PLCη

The PI-PLCη subfamily comprises two isoforms (η1 and η2). Their domain architecture aligns with the core PI-PLC structure, featuring a PH domain, an EF-hand domain, catalytic X-Y catalytic domains, and a C2 domain. Unlike PI-PLCζ, PI-PLCη isoforms retain the PH domain critical for phosphoinositide binding, while their EF-hand motifs confer increased sensitivity to Ca^2+^ compared to other PI-PLC isoforms. This structural configuration enables membrane association and Ca^2+^-dependent modulation of enzymatic activity [54].

PI-PLCη enzymes exhibit unique activation dynamics, responding to lower Ca^2+^ concentrations than other PI-PLCs, suggesting roles in fine-tuning Ca^2+^ homeostasis. While direct GPCR interactions remain under investigation, their structural homology to PI-PLCβ isoforms implies potential recruitment by Gαq/11 subunits [55].

PI-PLCη1 and η2 show predominant expression in neuronal and neuroendocrine tissues. In the brain, they likely modulate synaptic plasticity, neurotransmitter release, and Ca^2+^ oscillations. Neuroendocrine roles include regulating hormone secretion, as evidenced by their presence in pituitary and adrenal cell lines. Their Ca^2+^ sensitivity positions them as amplifiers of Ca^2+^-dependent signaling cascades in excitable cells [56].

## 4. Functions of PI-PLC Isozymes

**Figure 4:**
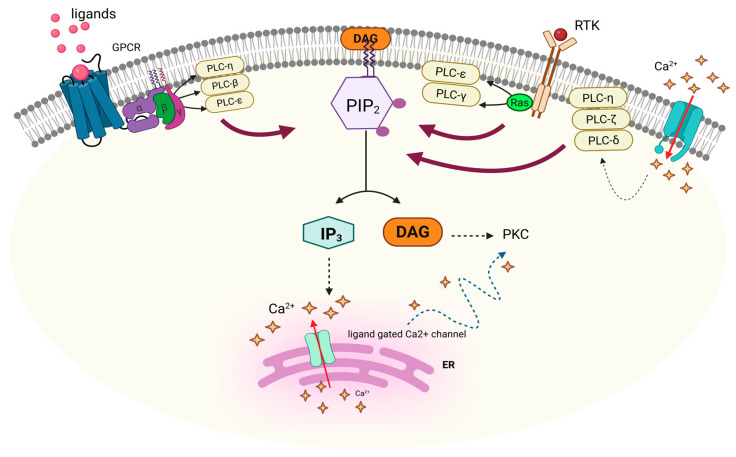
Function of PI-PLC isozymes. Created in BioRender. Nomikos, M. (2025). https://BioRender.com/ey8gepm (accessed on 26 May 2025).

The activation of PI-PLCs is primarily triggered by extracellular stimuli transmitted through multiple receptor types, which initiate distinct activation mechanisms depending on the PI-PLC isoform. Tyrosine kinase receptors (TKRs), integrin adhesion proteins, immune cell receptors such as Fc receptors, T-cell receptors, B-cell receptors, and GPCRs, and Ras GTPases modulate the activation of PI-PLCs, which in turn convey signals to other ligands, such as hormones and neurotransmitters (Figure 4) [14].

PI-PLCs cleave their primary phosphoinositide substrate, PI(4,5)P_2_, generating the messengers IP_3_ and DAG, which mediate signal transduction. Thus, activated PI-PLC lowers the available phosphoinositide in the membrane by cleaving it into other functional or signaling molecules. This mechanism is essential for a variety of cellular processes involved in the regulation of cell metabolism and growth, therefore further highlighting the critical role of PI-PLCs in multiple diseases and their therapeutic interventions.

Hormones, neurotransmitters, or growth factors bind to receptors on the cell surface (such as GPCRs or RTKs). This binding triggers intracellular signaling cascades that activate PI-PLC. GPCRs activate PI-PLCβ via G protein subunits, while RTKs activate PI-PLCγ through tyrosine phosphorylation.

Typically, upon activation by these upstream signals, PI-PLCs initiate the lipid signaling cascade, through interaction with inositol-containing phospholipids. PI-PLC catalyzes the hydrolysis of PI(4,5)P_2_ to generate IP_3_ (a Ca^2+^-mobilizing secondary messenger) and DAG (a PKC-activating secondary messenger). This reaction marks the initiation of the classic PI-PLC-PI(4,5)P_2_-IP_3_/DAG signaling pathway.

Due to its hydrophobic nature, DAG remains embedded in the membrane and activates PKC, which in turn regulates a broad array of cellular functions including growth, polarity, proliferation, and gene expression [57]. On the other hand, IP_3_, being water-soluble, diffuses into the cytoplasm and binds to IP_3_ receptors on the endoplasmic reticulum, consequentially inducing the release of Ca^2+^. The spike in intracellular Ca^2+^ levels regulates a plethora of enzymatic actions and signaling pathways. Interestingly, Ca^2+^ also binds to DAG to further enhance PKC activation. Besides activating PKC, DAG can also be cleaved into arachidonic acid, which serves as a precursor for important signaling molecules like leukotrienes and prostaglandins [58].

A comprehensive understanding of the mechanism of PI(4,5)P_2_ hydrolysis is essential to understanding the structure–function dynamics of the active sites, amino acid residues, co-factors, and other components that orchestrate this process.

The characteristic nature of PI-PLCs to transmit signals is tightly linked with their ability to hydrolyze phosphodiester bonds between the phosphate and glycerol groups of glycerophospholipids. This phosphodiesterase activity is Ca^2+^-dependent, as PI-PLC enzymes require a Ca^2+^ cofactor in their active site. The Ca^2+^ cofactor is ligated by the acidic residues of the enzyme—Asn312, Glu341, Asp343, and Glu390 [20]. Alterations in this acidic ridge of residues, for example, the mutation of Glu341 to Gly as shown by Cheng et al., can completely diminish the catalytic activity of PI-PLC [59].

The integration of the PI-PLC catalytic domain into the lipid bilayer to cleave membrane-bound PI(4,5)P_2_ is facilitated by a cluster of hydrophobic residues, Leu320, Tyr358, Phe360, Leu529, and Trp555, located nearby the active site [20]. These hydrophobic residues are conserved in all PI-PLC isozymes, suggesting their relevance in facilitating efficient catalytic activity of the enzyme. Moreover, the amino acid sequence of PI-PLC has the potential to strengthen the enzyme–substrate binding, by driving the formation of Van de Waals forces between the aromatic ring of Tyr551 and the PI(4,5)P_2_ inositol ring, leading to stable interactions. Additionally, the side chains of the residues Lys438, Lys440, Ser522, and Arg549 interact with the PI(4,5)P_2_ inositol ring at the 4′ and 5′ phosphorylated hydroxyl ends through hydrogen bonds and salt bridge interactions. It is due to these interactions that phosphoinositides phosphorylated at the 4′ and 5′ positions are preferred PI-PLC substrates, which explains why PI(4,5)P_2_ serves as the fundamental substrate of PI-PLC [20].

## 5. Regulation of Mammalian PI-PLC Isozymes

The presence of multiple PI-PLC isozymes provides differential means of regulating PI-PLC activity [12]. The regulation of mammalian PI-PLC isoforms involves the targeting of these enzymes to their substrates or activators via specific protein–protein and protein–lipid interactions mediated by the distinct regulatory domains, thus ensuring precise localization and activation of PI-PLC isozymes in response to receptor-induced changes within specific cellular environments [19]. PI-PLC enzymes mediate cellular signaling by converting extracellular stimuli into intracellular responses. Their control involves a complicated mix of protein–protein interactions, post-translational changes, and lipid modulation, demonstrating the complexity of cellular signaling networks [60].

PI-PLC isoforms’ unique regulatory mechanisms depend on their subtype. Several studies attribute the association of PI-PLCβ isozymes with heterotrimeric G protein subunits through interactions with GPCRs, coupled to both Gαq and Gαi family G proteins. Their hydrolysis products activate Ca^2+^ channels located on the endoplasmic reticulum (ER) membrane, leading to the release of Ca^2+^ from ER stores into the cytoplasm, thus influencing several cellular processes [19].

Several other studies have established the interlinked regulation of the γ-class subtype with tyrosine kinases. The nSH2 domain of PI-PLCγ interacts with phosphorylated tyrosine residues on RTKs, leading to the phosphorylation of a key tyrosine residue, Tyr783, in PI-PLCγ1, finally resulting in the cSH2 domain detaching from the catalytic site. These events confer the binding of PI(4,5)P_2_ to enable the activation of PI-PLCγ. Additionally, PI-PLCγ2 has the unique ability to interact with Rac through its split PH domain, to recruit it to the plasma membrane for activation. Both PI-PLCγ isoforms are further activated by PI(3,4,5)P_3_, which also aids their recruitment to the membrane. Thus, the intricate multidomain structure between the X and Y domains plays a crucial role in the activation and regulation of PI-PLCγ activity [19].

Contrastingly, PI-PLCδ isozymes lack unique domains; however, their PH domains, particularly PI-PLCδ1, exhibit a strong affinity for PI(4,5)P_2_. The binding of PI(4,5)P_2_ to these domains facilitates the translocation of PI-PLCδ to the plasma membrane, a crucial step for activation. Ca^2+^ additionally triggers the translocation of PI-PLCδ from the cytosol to the plasma membrane, leading to its activation. Based on this, it is also hypothesized that PI-PLCδ activation could be mediated by elevated Ca^2+^ levels due to the activity of other PI-PLC isoforms [19].

PI-PLCε activation occurs through the binding of its RA2 domain to the GTP-bound forms of Rap and Ras, resulting in distinct localization patterns wherein Ras directs PI-PLCε to the plasma membrane and Rap recruits it to the perinuclear region. The small GTPase RhoA binds to a specific region of the PI-PLCε Y domain, further activating PI-PLCε through Gα12/13-mediated RhoA activation via the Rho guanine nucleotide exchange factor (GEF), which further activates PI-PLCε in response to the ligands of Gα12/13-coupled receptors such as lysophosphatidic acid (LPA) and thrombin. Interestingly, the CDC25 homology domain in PI-PLCε acts as a GEF for Rap1, enhancing PI-PLCε’s lipase activity, thereby illustrating the complex regulation of PI-PLCε by various signaling pathways [61].

PI-PLCζ is activated by low concentrations of Ca^2+^ that approximate the resting cytosolic Ca^2+^ levels. This high sensitivity to Ca^2+^ is likely due to the EF-hand motif. Additionally, the electrostatic properties of the X-Y linker in PI-PLCζ differ from those in other PI-PLCs due to its positive charge, which may facilitate binding to the plasma membrane and interaction with the anionic substrate lipid PI(4,5)P2, suggesting that these activation mechanisms contribute to PI-PLCζ’s apparent constitutive activity [19].

Lastly, PI-PLCη exhibits high sensitivity to Ca^2+^ and appears to be activated by increases in intracellular Ca2+ levels. PI-PLCη2 is also activated by Gβγ, indicating that PI-PLCη2 activation can occur through stimulation of G protein-coupled receptors (GPCRs) [19]. The physiological roles of PI-PLCη remain largely unknown, and the absence of PI-PLCη2 does not result in any apparent abnormalities in mice, suggesting that PI-PLCη1 may compensate for the loss of PI-PLCη2 [62].

### 5.1. Protein–Protein Interactions

The onset of protein–protein interactions is central to leading PI-PLC isozymes to their biological destinations and regulating their activity [63]. Each PI-PLC isoform adapts and corresponds to different signals, subsequently interacting with several upstream signaling molecules, such as G proteins and receptor tyrosine kinases (RTKs), in turn initiating a cascade of events. For example, PI-PLCβ is activated by G protein-coupled receptors (GPCRs), whereas PI-PLCγ activation has been observed by RTKs. The activation of these two isoforms is suggested to be a result of extracellular stimuli [64]. Therefore, this highlights that this activation is not a one-size-fits-all process; rather, different isoforms respond to diverse signals, enhancing the accuracy of cellular responses.

### 5.2. Post-Translational Modifications

Post-translational modifications (PTMs) influence PI-PLC activity by impacting the structure, stability, and localization of the enzyme. These modifications also affect PI-PLC interactions and consequently cell signaling. The most frequent PTMs in PI-PLCs are primarily phosphorylation and, to a much lesser degree, ubiquitination. Among PI-PLC isoforms, PI-PLCγ is most notably prone to PTMs, whereas other isoforms may experience PTMs, but these are less integral to their regulation [18].

PLCγ is recruited to the membrane by binding of its SH2 domains to phosphorylated tyrosine residues on activated RTKs or adaptor proteins. Upon recruitment, the kinase phosphorylates specific tyrosines within the X-Y linker of PI-PLCγ (e.g., Tyr783 in PI-PLCγ1 and Tyr759 in PI-PLCγ2). Phosphorylation of these tyrosine residues, particularly Tyr783, disrupts the interaction between the C-terminal SH2 domain and the XY catalytic domain, releasing the active site and relieving autoinhibition. The conformational change induced by phosphorylation allows PI-PLCγ to access and hydrolyze its substrate [65].

### 5.3. Lipid Modulation of PI-PLC Activity

PLC activity is tightly regulated by the availability, spatial organization, and dynamic metabolism of membrane lipids. The lipid content of cellular membranes is critical to regulating PI-PLC activity, as these enzymes prefer phosphatidylinositol (PI) lipids for their function. Any variation in the lipid levels and their environment significantly affects their activity and localization within the cell. For instance, fluctuating levels of PI(4,5)P_2_ directly impact their catalytic activity [66].

Furthermore, DAG and IP_3_ actively participate in a feedback loop, to modulate enzyme activity. DAG, for example, can activate PKC, thus driving downstream signaling cascades. Therefore, understanding the lipid modulation of PI-PLC activity is imperative to unraveling the complexities of cellular signaling because it underscores the interrelated nature of lipid and protein signaling networks [67].

### 5.4. Integration of Regulatory Mechanisms

The complex interplay of several mechanisms that govern the regulatory aspect of PI-PLCs is far from merely being a linear process [56]. Protein–protein interactions, post-translational changes, and lipid modulation are all intimately linked, resulting in a dynamic regulatory network. A specific protein–protein interaction, for example, may trigger phosphorylation events, thereby altering the lipid environment and PI-PLC activity. In line with this, studies have shown that cells involved in these regulatory mechanisms respond to a multitude of extracellular signals and influence the functioning of PI-PLCs. Moreover, any deregulation within these systems can lead to a variety of illnesses, underlining the therapeutic potential of targeting PI-PLC-mediated signaling pathways [56]. Exploring the interwoven characteristics of these signaling cascades, in line with identifying treatment techniques, could pave the way for potential therapeutic strategies to combat the multifaceted complexities of PI-PLCs.

## 6. Implications of PI-PLCs in Health and Disease

**Figure 5:**
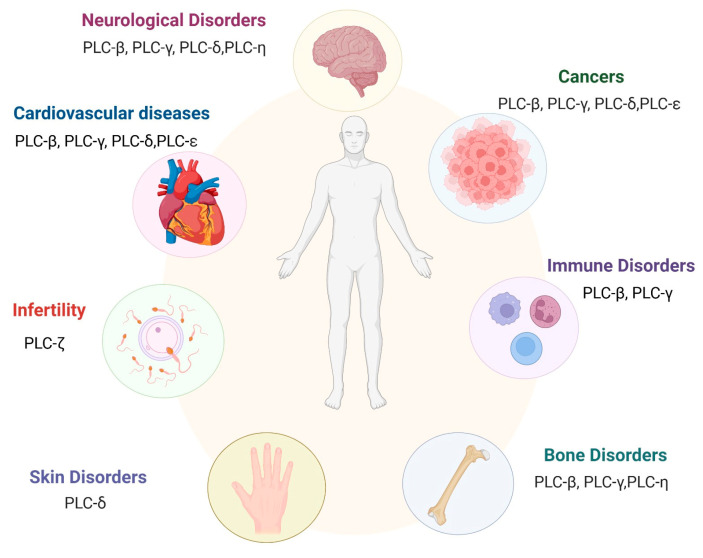
Correlation of PI-PLC isozymes with human pathologies. Created in BioRender. Nomikos, M. (2025). https://BioRender.com/v7fmwb8 (accessed on 26 May 2025).

Phosphoinositides constitute a mere 0.2–1% of total membrane phospholipids yet exert a disproportionate influence on cellular physiology and signaling [68]. In healthy individuals, phosphoinositide hydrolysis by PI-PLCs maintains metabolic equilibrium, whereas dysregulation of PI-PLC-mediated phosphoinositide metabolism is strongly implicated in the pathogenesis of multiple diseases (Figure 5) [56].

### 6.1. PI-PLC and Cancer

Phosphoinositide signaling via PKC activation and Ca^2+^ mobilization plays a vital role in maintaining fundamental cellular processes such as cell migration, survival, and differentiation, all of which are of high relevance to cancer development and metastasis. PI-PLC isozymes regulate cell proliferation, growth, and survival, by coordinating multiple pathways, most notably the PI3K/Akt/mTOR pathway, RAS/RAF/MAPK/ERK pathway, and JAK/STAT pathway. Disruptions in PI-PLC-mediated regulation of these pathways are strongly associated with aberrant cell proliferation and cancer pathogenesis. Studies have identified specific PI-PLC isozymes as key contributors to tumorigenesis across multiple cancer types. On the contrary, PI-PLCζ and PI-PLCη, have not yet been directly linked to cancer [56].

#### 6.1.1. PI-PLCβ and Cancer

Abnormalities within PI-PLCβ1 and the PI3K/Akt/mTOR pathway have been linked to myelodysplastic syndrome (MDS), a pathological condition characterized by hematopoietic stem cell mutations, amongst which 30% of MDS patients were found to progress to acute myeloid leukemia (AML) [56]. Discovering the presence of a certain monoallelic deletion of the PI-PLCβ1 gene was correlated to the progression of MDS to AML, paving the way for newer investigations. Poor clinical outcomes in MDS patients additionally elevated the risk of AML onset [69]. These findings were further supported by a study investigating MDS clinical outcomes, under azacitidine treatment, which is a DNA methyltransferase inhibitor aiding in the delay of AML development. PI-PLCβ1 mRNA expression was used as an indicator of azacitidine effectiveness. Higher levels of the mRNA reflected better clinical outcomes, whereas lower levels indicated worse clinical outcomes [70]. Additional findings from this study highlighted the interlink between fluctuating PI-PLCβ1 expression and Akt activity, which could impact the cell cycle in MDS cells, ultimately inhibiting apoptosis while enhancing MDS cell survival.

It has been identified that PI-PLCβ3 has a tumor suppressor function, notably in hematologic malignancies [71,72]. PI-PLCβ isoform was found to function not enzymatically but rather as an adaptor mediating tumor suppression. Mice deficient in PI-PLCβ3 are prone to developing neoplastic diseases, such as myeloproliferative neoplasms, chronic lymphocytic leukemia, acute lymphoblastic leukemia, and some lymphomas. This neoplasm suppressor function was attributed to the carboxyterminal of the PI-PLCβ3, which acts as an adaptor to mediate the inactivation of Stat5, a critical promoter of cell proliferation and survival, inhibiting neoplastic growth. PI-PLCβ assembles Stat5 and the protein tyrosine phosphatase, SHP-1, with a newly discovered multimolecular signaling complex called the SPS complex. SHP-1 can dephosphorylate Stat5, thereby inactivating its growth-inducing function. Lyn, a component of the SPS complex, can phosphorylate SHP-1 at tyrosine residues 536 and 564, which enhance SHP-1 phosphatase activity and regulate its interaction with Stat5, respectively. This SPS-dependent activation of SHP-1, following PI-PLC-β-mediated configuration, is crucial for maximizing SHP-1 activity toward its substrate [71].

#### 6.1.2. PI-PLCγ and Cancer

PI-PLCγ is also associated with multiple cancers such as gliomas and colorectal and gastric cancers [73,74]. A study revealed elevated levels of PI-PLCγ expression in tissues of squamous cell carcinoma in comparison to the surrounding normal epithelial tissue. Understanding the mechanism by which PI-PLCγ1 induces SCC cell mitogenesis can be achieved by comparing PI-PLCγ1 knockdown with PI-PLCγ1 enzymatic lipase activity inhibition. While inhibiting PI-PLCγ1 activity did not affect mitogenesis, PI-PLCγ knockdown blocked EGFR-mediated SCC mitogenesis, suggesting that the role PI-PLCγ1 plays in cancer cell mitogenesis is independent of its enzymatic activity and could be via SH domain-related protein interactions [73].

Recent studies established a link between the aggressiveness of oral SCC and elevated expression levels of PI-PLCγ levels, therefore consolidating the close association between PI-PLCγ and cancer [75].

#### 6.1.3. PI-PLCδ and Cancer

PI-PLCδ was also found to be involved in several cancers. Elevated levels of PI-PLCδ in breast cancer patients were significantly associated with a short disease-free survival [76]. The study examined the expression of different PI-PLC isoforms in breast cancer tissue. The results showed higher expression levels of PI-PLCβ, PI-PLCδ, and PI-PLCε in cancerous versus non-cancerous tissue. Conversely, PI-PLCγ had lower levels in cancerous breast tissues as compared to normal tissues. The study further applied Kaplan–Meier survival analysis to assess the role of PI-PLC isozymes on patients’ outcomes in cases of breast cancer. It was found that, in patients with elevated levels of PI-PLCδ, the average disease-free survival was shorter (96.7 months) compared to patients with lower levels of PI-PLCδ (137.4 months). While the study affirmed that PI-PLCδ has a strong association with breast cancer prognosis, the rest of the PI-PLC isozymes were not found to impact disease-free survival [76].

#### 6.1.4. PI-PLCε and Cancer

PI-PLCε was found to be affected differently in different types of cancer, wherein increased levels of PI-PLCε1 mRNA and protein levels were correlated with gastric cancer [77], whereas the downregulation of PI-PLCε1 and PI-PLCδ1 was associated with colorectal cancer [78]. Additionally, PI-PLCε also has a tumor suppressor role via the RAS pathway, a cascade involved in cancer progression [79].

### 6.2. PI-PLC and Immune Disorders

#### 6.2.1. PI-PLCβ and Immune Disorders

PI-PLCβ isoforms in immune physiological processes are vital for the activation and differentiation of innate and adaptive immune cells that control innate and adaptive immunity [71,80]. In a recent study, it has been shown that PI-PLCβ3 is the main functional PLCβ isoform in murine macrophages. Macrophages from PI-PLCβ3-deficient mice were highly sensitive to apoptosis inducers. In an atherosclerosis model (apoE-deficient mice), PLCβ3 deficiency led to fewer total macrophages and increased macrophage apoptosis within atherosclerotic lesions. The findings suggest that PI-PLCβ3 activity promotes macrophage survival within atherosclerotic plaques, and targeting PI-PLCβ3 could be a novel strategy for reducing atherosclerosis [81].

Another study emphasized the role of T-cells, indicating that the loss of PI-PLCβ2 and PI-PLC-β3 severely impaired T-cell migration. SDF-1α/CXCL12), a chemokine, is the sole ligand of CXCR4. In PI-PLCβ2−/−, a PI-PLCβ3−/− double knockout (dko) lymphocyte, blocking Ca^2+^ influx leads to disrupted T-cell migration. The lack of Ca^2+^ signals, controlled by SDF-1α, in the knockout cells, suggested that the defect in migration was due to an impaired ability to increase intracellular Ca^2+^. Additional findings have shown that inhibiting PLC functioning in turn inhibits T-cell migration via CXCR3 [82].

Neutrophils, a class of innate immune cells, are also regulated by PI-PLCβ isoforms. Two genetic loci, discovered in a genome-wide study, exhibited variations in neutrophil count [83]. One of these genetic loci was found in the *PI-PLCB4* gene, while the other was found in the *PSMD3–CSF3* gene (rs4794822 in *PSMD3–CSF3* and rs2072910 in *PI-PLCB4*). Participants homozygous for the neutrophil-increasing alleles of these two genes (T alleles for rs4794822 and rs2072910) had 1.17-fold higher neutrophils as compared to those homozygous for the neutrophil-decreasing alleles of these two genes (C alleles for rs4794822 and rs2072910). The findings therefore underline that *PSMD3–CSF3* and *PI-PLCB4* genes may possibly be involved in regulating the production of neutrophils [83].

Furthermore, studies have also established that PI-PLCβ contributes to adaptive immune processes such as T-cell and B-cell functions. The loss of PI-PLCβ2 and PI-PLCβ3 was also shown to impair T lymphocyte migration and chemotaxis [71].

#### 6.2.2. PI-PLCγ and Immune Disorders

Mutations in PI-PLCγ2 were found to be associated with a complex immune condition known as PLAID (PI-PLCγ2-associated antibody deficiency and immune dysregulation—PLCG2 GOF). This condition is a result of a germline deletion mutation and is dominantly inherited [84]. This is accompanied by autoimmunity as well as immune dysfunction [85]. The mutated PI-PLCγ2 variant produces constitutively active PI-PLCγ2. Despite the observed enzyme hyperactivity, the condition is marked by reduced Ca^2+^ flux in both B and NK cells, decreased ERK (extracellular signal-regulated kinase) phosphorylation, and decreased degranulation in B cells and NK cells, respectively. It has been hypothesized that a constitutive increase in enzymatic function, coupled with diminished downstream signaling, can be possibly explained by the depletion of PI(4,5)P_2_ due to overactive enzyme function or the downregulation of downstream signaling by feedback inhibition. Interestingly, an external environmental factor, in this case, cold temperatures, seems to influence PI-PLCγ2 downstream signaling and induce degranulation of mast cells, manifesting clinically as cold-induced urticaria in PLAID patients [85].

Another interesting mutation of the PI-PLCγ isozyme also affects PI-PLCγ2. However, in this case, the inherited mutation is a Ser707Tyr substitution, leading to autoinflammatory PLAID (APLAID) [86]. While PLAID patients suffer from both immune dysfunction and autoimmunity, APLAID patients exhibit pronounced autoinflammatory disease and do not experience the cold-induced urticaria observed in PLAID patients. This substitution mutation leads to higher Ca^2+^ flux instead, alongside ERK phosphorylation in B cells. While both mutations result in an overactive enzyme, the substitution mutation is hypothesized to yield a slightly more active PI-PLCγ2 enzyme than the deletion mutation, which could account for increased downstream signaling and irresponsiveness to feedback inhibition [85]. Despite this variation in APLAID- and PLAID-causing mutations, both mutations similarly affect a distinct downstream pathway that induces a characteristic loss of class-switched memory B-cells. Investigating the commonality in their outcomes could pave the way to giving a broader perspective on PI-PLCγ2 mutations in immune regulation.

### 6.3. PI-PLC and Cardiac Disorders

#### 6.3.1. PI-PLCβ and Cardiac Disorders

Amongst the well-known PI-PLC isozymes, PI-PLCβ has been associated specifically with normal cardiac physiology as well as cardiac pathologies. PI-PLCβ isozymes regulate cardiac muscle contraction via Ca^2+^ signaling. As the signaling cascade progresses, Ca^2+^ is released from intracellular stores eventually, enhancing the force of cardiac muscle contraction crucial for cardiac function maintenance and pumping efficiency [87]. Regarding the relevance of two PI-PLCβ1 splice variants, PI-PLCβ1a and PI-PLCβ1b, to impaired cardiac tissue, using mouse models has shown elevated activation of PI-PLC-β1b, and not PI-PLCβ1a, inducing loss of contractility, which could be reversed via PKCα inhibition. PI-PLCβ1b-induced activation of PKCα leads to the dephosphorylation of phospholamban (activation of phospholamban) and consequently the depletion of Ca^2+^ intracellular stores, thus emphasizing the importance of modulating PI-PLCβ1b activity in cardiac muscle pathology [39].

PI-PLC is also implicated in another condition—cardiac hypertrophy. Under volume or pressure overload stimuli, the cardiac myocardium adapts by enlarging itself, leading to hypertrophy and consequently congestive heart failure. Several pro-hypertrophic stimuli were found to activate PI-PLCβ isoforms, such as angiotensin II (ANG II), phenylephrine (PE), vasopressin, and α1-adrenergic agonists [87]. PE stimulation, a useful drug for blocking β-adrenergic receptors (ARs), elevated PI-PLCβ1b expression in neonatal ventricular rat cardiomyocytes. Those stimuli inciting hypertrophic response were correlated with PI-PLCβ1b expression and hypertrophic cellular changes, including a higher cell area and a high protein/DNA ratio [88].

Additionally, GPCR signaling is also linked to PI-PLC activation and has been deemed pivotal in hypertrophic myocardial growth [89]. Myocardium hypertrophy has been shown to worsen with elevated Gq activity and improve in the case of Gq inhibition. In line with this, it was also found that the Gq/PI-PLCβ pathway, which increases Ca^2+^ levels inside the cells, prompting PKC activation and the calcineurin/NFAT pathway (calcineurin/nuclear factor of activated T-cell pathway), is also closely tied to hypertrophic responses [90].

Gαq mutants with impaired signaling through PI-PLCβ are unable to effectively induce muscle hypertrophy [91]. The Gq activity causing myocardium hypertrophy is specifically mediated by the PLCβ1b splice variant. Using a modified 32-aa C-terminal PI-PLCβ1b peptide, it was observed that this peptide competes with PI-PLCβ1b, prevents its binding with the sarcolemma, and consequently blocks its association with Gq, in turn blocking a hypertrophic response. This offers a promising therapeutic approach to managing hypertrophy by inhibiting PI-PLCβ1b and improving disease outcomes [91].

Recent studies also elucidate the significance of PI-PLC signaling in insulin-dependent cardiomyopathy, in connection with the disrupted α1-adrenergic receptor (α1-AR). The receptor influences PI-PLCβ isozymes via Gqα. Notably, PI-PLCβ3 signaling in heart cells is altered, in a way that impacts its distribution and activity, resulting in reduced IP_3_ levels, which weaken the contraction of the isolated papillary muscle in response to α1-adrenergic stimulation. A further decrease in DAG also affects several cellular processes including Ca^2+^ signaling [92].

#### 6.3.2. PI-PLCε and Cardiac Disorders

In recent years, several studies have demonstrated the association of PI-PLCε1 in cardiovascular diseases. The distinctive RA-1 domain present in this PI-PLC isoform allows it to interact with muscle-specific A-kinase anchoring protein (mAKAP) on the perinuclear membrane, triggering the hydrolysis of PI(4)P in cardiomyocytes, thus initiating signal transduction processes involved in cardiac functioning. Activated PI-PLCε1 results in the production of DAG and IP_3_, which are pivotal for their roles in regulating key signaling pathways associated with cardiac disorders. Both secondary messengers interfere with normal Ca^2+^ release, affecting Ca^2+^ sensitivity and contractility. Additionally, when DAG activates the PKC signaling cascade, it impacts several other pathways such as ERK1/2 signaling and Akt-mTOR signaling, leading to chronic vascular complications, cardiac hypertrophy, heart failure, and myocardial ischemia/reperfusion (I/R) injury [93].

### 6.4. PI-PLC and Neurological Disorders

#### 6.4.1. PI-PLCβ and Neurological Disorders

PI-PLCs are abundantly expressed across different areas of the brain and are therefore linked to numerous brain disorders. PI-PLCβ1, specifically, has been localized in parts such as the hippocampus and cerebral cortex. A study in PI-PLCβ1 knocked-out mice as well as in human patients found a correlation between PI-PLCs and neurological disorders such as epilepsy, schizophrenia, and bipolar disorders. Likewise, PI-PLCγ1 influences neuronal cell migration and neuroplasticity, therefore linking it to Alzheimer’s disease, Huntington’s disease, and a few others [94].

#### 6.4.2. PI-PLCγ and Neurological Disorders

In a genome-wide association study (GWAS), it was discovered that there was a rare genetic mutation of the PI-PLCγ2 gene, wherein proline was substituted with arginine on the 522nd codon (Pro533Arg). This was linked to a decreased risk of Alzheimer’s disease (AD). The Pro522Arg mutation was also found to reduce the risk of other dementias such as Lewy body dementia (LBD) and frontotemporal dementia. However, this variant did not prevent the onset of Parkinson’s disease, amyotrophic lateral or multiple sclerosis, and supranuclear palsy [95].

PI-PLCγ2, predominantly present in microglia, the resident immune cells in the brain, was found to be also implicated with AD [96]. Microglia-like cells derived from genetically modified human-induced pluripotent stem cells exhibited activation of myeloid cell 2 (TREM2) receptors, which prompted signaling through PI-PLCγ2. This signaling pathway is involved in microglial cell activity including cell survival, phagocytosis, the processing of neuronal debris, and the regulation of lipid metabolism. Thus, the absence of TREM2 or PI-PLC signaling results in dysregulated transcription, a characteristic of the disease phenotypes. PI-PLCγ2 is also identified as a mediator of inflammatory responses downstream of Toll-like receptors involving TREM2 and other mediators, which may explain the shift to the pathologic microglial state characteristic of neurodegenerative disease [96,97,98].

### 6.5. PI-PLCζ and Infertility

PI-PLCs have also been implicated for their role in infertility. The PI-PLCζ isoform has been specifically characterized as being exclusively sperm-specific and playing a crucial role in oocyte activation post-fertilization. Egg activation physiologically follows sperm–egg fusion and is vital for liberating the egg from meiotic arrest to resume the second meiotic division, preventing polyspermy by initiating the cortical reaction, and stimulating the maternal-to-zygotic transition by translating maternal mRNAs [99,100]. Therefore, several events are essential for gamete fusion and eventually embryonic development, all of which are primarily influenced by Ca^2+^ release [101,102].

Ca^2+^ transients via IP_3_ pathway signaling consistently appear in egg activation across several species investigated so far [102]. In line with this, experimental studies in mice and hamster eggs exhibited dysregulated IP_3_ receptor activity, in response to lower levels, downregulation, or receptor inhibition blockage, thus disrupting Ca^2+^ repetitive spikes required for oocyte activation. This finding provided further evidence supporting the involvement of IP_3_ signaling in fertilization [101].

Subsequent steps of the PI-PLC pathway include PI(4,5)P_2_ hydrolysis and IP_3_-mediated generation of Ca^2+^. The rise in Ca^2+^ is a critical signal for fertilization and also modulates the cytoplasmic conditions necessary for oocyte activation. The spike in Ca^2+^ is observed as a series of oscillations in mammals [101]. Since the PI-PLCζ-generated Ca^2+^ transients are crucial for embryogenesis, abnormalities within mRNA sequence, expression level, or function are implicated with infertility.

Previous studies have already demonstrated that immunodepletion of PI-PLCζ was the causative factor for the absence of Ca^2+^ oscillations necessary for egg activation [49]. Multiple studies on sperm from infertile men have revealed the inability of sperm to induce Ca^2+^ oscillations or otherwise, an abnormal pattern characterized by reduced amplitude and frequency. Hence, the absence, low expression levels, and abnormal localization patterns of PI-PLCζ in sperm are closely associated with male infertility [102].

### 6.6. PI-PLCβ and Bone Diseases

In bone biology, specific PI-PLC isoforms modulate osteoblast differentiation, osteoclast activity, and extracellular vesicle-mediated signaling, with dysregulation implicated in diseases such as osteosarcoma and metabolic bone disorders.

PI-PLCβ1, for instance, drives mesenchymal stem cell commitment to osteogenic lineages, a process essential for bone formation and repair. Conversely, aberrant expression of PI-PLC isoforms, particularly in osteosarcoma, correlates with tumor aggressiveness and metastatic potential. Osteosarcoma-derived extracellular vesicles package multiple PI-PLC isoforms, which may condition surrounding cells to adopt tumor-like phenotypes, potentially serving as biomarkers for disease progression. Additionally, PI-PLCη2, localized near osteoblast membranes, responds to Ca^2+^ fluctuations and may coordinate bone remodeling by modulating intracellular Ca^2+^ stores through IP_3_-mediated mechanisms.

Recent studies highlight PI-PLCβ4 as a regulator of osteoclast differentiation via RANKL signaling, linking its activity to bone resorption disorders [103]. In osteosarcoma, PI-PLC-dependent Ca^2+^ signaling anomalies contribute to cell survival and proliferation, with specific isoforms influencing PI(4,5)P_2_ metabolism and downstream pathways critical for tumorigenesis [104]. These findings underscore the dual role of PI-PLCs in maintaining bone health and driving pathology, positioning them as potential therapeutic targets. For instance, targeting PI-PLC-mediated EV signaling in osteosarcoma could disrupt tumor–microenvironment interactions, while modulating PI-PLCβ4 activity might mitigate excessive osteoclast activation in osteoporosis.

### 6.7. I-PLCδ and Skin Disorders

Mutations or loss of function in the *PI-PLCD1* gene are strongly associated with several skin and nail disorders. Most notably, PI-PLCδ1 mutations cause hereditary leukonychia, a rare condition characterized by white discoloration of all fingernails and toenails, due to abnormal keratinization in the nail matrix. PI-PLCδ1 is also highly expressed in keratinocytes and other skin tissues, and its deficiency can lead to features of interleukin-17-associated inflammatory skin diseases, increased cutaneous inflammation, and abnormal hair formation. Additionally, certain mutations in PI-PLCδ1 have been linked to the development of trichilemmal cysts and pilomatricoma-like lesions, further highlighting its critical role in skin and appendage health [105].

These findings shed light on the multifaceted role that PI-PLCs play under several pathological conditions discussed above. In addition to their mechanistic roles in cell signaling and contribution towards disease progression, PI-PLCs also hold potential as disease biomarkers. In cancers, for example, they contribute towards tumor suppression as well as progression, as shown in the case of downregulated PI-PLCβ1 in myelodysplastic syndrome, the tumor suppressive behavior of PI-PLCβ3 in leukemias, and the overexpression of PI-PLCγ1 in squamous cell carcinoma. Beyond oncology, under neurological conditions, several studies reveal that PI-PLCs critically regulate Ca^2+^ signaling, and therefore, their expression levels are linked to prevalent conditions such as Alzheimer’s and epilepsy. Additionally, PI-PLCβ1b and PI-PLCε also exacerbate the effects of cardiac hypertrophy and cardiac remodeling, therefore linking PI-PLCs to cardiovascular disease interplay. PI-PLCζ stands out for its role in the fertilization process, making it a unique diagnostic and therapeutic biomarker specifically in male infertility.

## 7. Therapeutic Potential of Targeting PI-PLCs

Targeting PI-PLC isozymes holds therapeutic promise for a range of disorders due to the central role of PI-PLC signaling in cellular physiology and disease pathogenesis. Isozyme-specific inhibitors and recombinant supplementation are essential to minimizing side effects and offering more precise intervention.

### 7.1. U73122 in Management of Inflammatory Disorders

U73122 is a widely used and well-characterized pharmacological inhibitor of PI-PLC, making it an essential tool for dissecting PI-PLC-dependent signaling pathways in cellular studies. Its ability to inhibit IP_3_ synthesis and downstream Ca^2+^ signaling has allowed researchers to clarify the role of PI-PLC in cell adhesion, differentiation, and other physiological responses. In studies examining promonocytic cell adhesion and differentiation, U73122’s dose-dependent inhibition of PI-PLC activity has been critical for demonstrating that PLC signaling is necessary for the activation of adhesion-related proteins like Pyk2 and paxillin. However, it is important to note that U73122 can also inhibit other cellular targets, such as the sarcoplasmic/endoplasmic reticulum Ca^2+^ ATPase (SERCA) pump, independent of its effect on PI-PLC, which may complicate data interpretation in some contexts. Despite these caveats, U73122 remains a valuable and potent inhibitor for probing the significance of PLC in a wide range of biological processes [106].

There is also a link between PI-PLCγ and its inhibitor U73122 in Graves orbitopathy (GO). GO results from an autoimmune condition, Graves’ disease, where antibodies attack the thyroid-stimulating hormone receptor (TSH-R) and induce hyperthyroidism. In 40% of cases, an ocular manifestation of the disease occurs, resulting in GO [107]. Conventional interventions include corticosteroids, external beam radiation, and immunosuppressive agents, yet current treatment is very effective in addressing the underlying pathological changes in the orbital tissues [108]. Roh et al. (2023) [107] found elevated PLC-γ1 and PLC-γ2 expression in GO patients’ orbital tissue as compared to normal tissues. The PLC-specific inhibitor U73122 induced anti-inflammatory changes in these cells and suppressed proinflammatory molecules, such as IL-6, IL-8, monocyte chemoattractant protein-1, cyclooxygenase-2, and intercellular adhesion molecule-1 (ICAM-1). In addition to this, Akt and p38 kinase phosphorylation was inhibited in GO fibroblasts, therefore suggesting the therapeutic potential of U73122 to target the PI-PLCγ-mediated pathogenesis of GO [107].

Overall, in further research on the complexity of PI-PLC signaling with regards to inflammatory responses, using animal models and clinical trials is needed as PI-PLC enzymes are promising targets for developing novel anti-inflammatory treatments.

### 7.2. U73122 in Management of Cancer

One of the leading causes of cancer-related mortality is metastasizing tumors that show limited response to several therapies. Based on this, there have been studies that have delved into the use of U73122 in the treatment of various cancers, owing to its ability to reverse PI-PLC-mediated cancer growth. The potential of U73122 in osteosarcoma treatment was highlighted using the MG-63 osteosarcoma cell line [109]. U73122 was found to remarkably abrogate MG-63 cell growth while altering the gene expression patterns and localization of some PI-PLC isoforms. The interplay linking PI-PLC inhibition and osteosarcoma suppression could occur through regulating ezrin, a protein belonging to the ezrin–radixin–moesin (ERM) family. Ezrin, implicated with osteosarcoma progression and metastasis, was found to be regulated by PI(4,5)P_2_ levels, under PI-PLC enzyme activity. These outcomes suggest that U73122 affects PI-PLC isoform expression differently to induce growth inhibition in MG-63 osteosarcoma. Remarkably, U73122 decreased the expression of PI-PLC isoforms associated with cancer progression and increased expression of PI-PLCβ1, which is thought to promote differentiation and apoptosis. This dual effect underscores the potential of U73122 in meticulously modifying PI-PLC activity to hinder osteosarcoma growth and enhance therapeutic outcomes [109]. However, further studies including in vivo models and clinical validation are required to confirm these preliminary findings and assess safety and specificity [110].

Inhibitors of PI-PLCs may mitigate the adverse effects of chemotherapy in cancer treatment. Doxorubicin, a cytotoxic chemotherapeutic agent used to treat solid neoplasia and hematologic malignancies, can irreversibly induce cardiotoxicity. This condition is characterized by elevated plasma levels of endothelin-1 (ET-1), its receptors, and PI-PLCβ2, leading to cardiomyocyte remodeling, marked by left ventricular dysfunction, dilation, and cardiomyocyte injury [87]. ET-1 receptor inhibitors reverse upregulation and impart a protective role against doxorubicin-induced myocardial hypertrophy [111]. Likewise, another study using the PI-PLC inhibitor U73122 was able to counteract the elevated PI-PLCβ expression induced by NE and Ang II, providing a cardioprotective effect [112]. Nevertheless, U73122 also exhibits off-target effects and limited in vivo data, requiring caution and additional investigation to establish clinical relevance.

Further research is needed to investigate the potential of inhibiting PI-PLCβ2 with PI-PLC inhibitors like U73122, in alleviating doxorubicin-induced cardiotoxicity. Analyzing these mechanisms through further studies could uncover targeted treatment strategies for cancer management while taking into account treatment efficacy, minimizing side effects, and improving patient care outcomes.

### 7.3. NSC768313 in Management of Cancer

NSC768313, a thieno [2,3-b]pyridine derivative, has emerged as a promising anticancer agent with a unique mechanism linked to PI-PLC modulation. Preclinical studies demonstrate that NSC768313 disrupts cancer cell proliferation by targeting PI-PLC isoforms, particularly PI-PLCδ, thereby suppressing the hydrolysis of PI(4,5)P_2_ into DAG and inositol trisphosphate IP_3_. This inhibition impairs downstream PKC activation and Ca^2+^ signaling, which are critical for mitogenic and survival pathways in tumors. Additionally, NSC768313 induces G_2_/M cell cycle arrest and membrane blebbing in breast cancer cells, such as MDA-MB-231, through tubulin dysregulation, without causing DNA damage, and synergizes with taxanes like paclitaxel by enhancing cytotoxicity via dual targeting of PI-PLC-mediated signaling and microtubule stability. In vitro studies reveal potent growth inhibition across NCI60 cancer cell lines, with low micromolar IC_50_ values, and aberrant cytokinesis linked to disrupted cytoskeletal regulation. Pharmacokinetic data in mice show a plasma half-life of ~4 h, supporting its potential for combination therapies.

The Ca^2+^-sensitive PI-PLCδ isoform, with its EF-hand domains and conserved catalytic residues (e.g., His-311, Glu-341), is a likely target due to its role in Ca^2+^ oscillations during cell division, and NSC768313’s interaction with these structural motifs may explain its specificity. By sparing receptor-coupled PI-PLC-β/γ isoforms, NSC768313 offers selective inhibition of PI-PLCδ’s PI(4,5)P_2_ hydrolysis, minimizing off-target effects. Its synergy with taxanes underscores the therapeutic potential of targeting PI-PLC isozymes to overcome chemotherapy resistance, positioning NSC768313 as a novel candidate for further development in precision oncology strategies [113,114].

### 7.4. Recombinant PI-PLCζ in the Treatment of Male Infertility

The alarming prevalence of male infertility continues to be poorly explained in many cases, to date. While the discovery of ART methods like ICSI or IVF was a breakthrough in successfully treating some infertility cases, the underlying causes and challenges in severe cases of male infertility continue to persist [115].

PI-PLCζ is extensively studied owing to its role in oocyte activation and its therapeutic potential in the treatment of male infertility. PI-PLCζ induces Ca^2+^ oscillations necessary for egg activation, the absence of which could result in oocyte activation deficiency (OAF) or failure. While some treatment strategies for replacing the PI-PLCζ function have been developed, for example, using Ca^2+^ ionophores, these fall short in generating the characteristic Ca^2+^ oscillation pattern in mammalian eggs. Typically, Ca^2+^ ionophores solely produce a single Ca^2+^ transient, which can increase the success rates of fertilization and implantation following intracytoplasmic sperm injection (ICSI). Yet, this approach raises concerns regarding subsequent embryo development, the potential of teratogenicity and cytotoxicity, and epigenetic complications [116]. These challenges have paved the way for the use of recombinant PI-PLCζ as a prominent and effective approach in the treatment of OAF.

The findings demonstrate the effectiveness of recombinant PI-PLCζ protein with a maltose-binding protein (MBP) fusion tag in generating potent Ca^2+^ oscillations following microinjection into mouse eggs. These oscillations were analogous to the oscillations induced by microinjecting a native sperm extract. To assess the safety of the treatment, recombinant PI-PLCζ was injected into a chick embryo model and monitored for potential cytotoxicity. The results showed normal embryonic viability of the embryos, providing preliminary evidence that MBP-PI-PLCζ protein could be potentially safe in human embryos. To elucidate the safety and therapeutic potential of recombinant PI-PLCζ, comprehensive investigations are needed to understand the promising clinical applications that could follow [116].

## 8. Conclusions

PI-PLCs metabolize phosphoinositides, primarily PI(4,5)P_2_, to transmit extracellular signals and mediate signal transduction, thus participating in a variety of cellular functions. This review has underscored the structural features of PI-PLC isozymes as well as their function and regulation mechanisms, to understand their close involvement in various physiological and pathological conditions. The dysregulation or absence of PI-PLCs is the culprit of many disorders, including cancer, immune conditions, neurological diseases, cardiac disorders, and male infertility. Thus, targeting these enzymes by using small molecules or supplementing recombinant PI-PLCs is a promising therapeutic approach to many health conditions including cancer and male infertility.

## Figures and Tables

**Table 1:** Summary of the different PI-PLC isoforms, their tissue distribution, and their activation mechanisms.

Isoform	Main Tissue Distribution	Activation Triggers/Mechanisms
PLCβ	Ubiquitous (brain, heart, etc.)	GPCRs via Gαq and Gαi (directly by Gαq; indirectly via βγ subunits)
PLCγ	Widespread (immune cells, etc.)	Receptor tyrosine kinases (RTKs; e.g., EGFR, PDGFR)
PLCδ	Ubiquitous	GPCRs (indirectly via Ca^2+^), increased cytosolic Ca^2+^
PLCε	Heart, brain, others	Ras, Rap GTPases, Gα12/13, Gβγ, RTKs
PLCζ	Sperm	Intracellular Ca^2+^
PLCη	Brain, others	GPCRs
